# Association of Chemerin Plasma Concentration With Risk of Colorectal Cancer

**DOI:** 10.1001/jamanetworkopen.2019.0896

**Published:** 2019-03-22

**Authors:** Fabian Eichelmann, Matthias B. Schulze, Clemens Wittenbecher, Juliane Menzel, Cornelia Weikert, Romina di Giuseppe, Ronald Biemann, Berend Isermann, Andreas Fritsche, Heiner Boeing, Krasimira Aleksandrova

**Affiliations:** 1 Senior Scientist Group Nutrition, Immunity and Metabolism, Department of Nutrition and Gerontology, German Institute of Human Nutrition Potsdam–Rehbrücke (DIfE), Nuthetal, Germany; 2 German Centre for Cardiovascular Research (DZHK), Berlin, Germany; 3 Department of Molecular Epidemiology, German Institute of Human Nutrition Potsdam–Rehbrücke (DIfE), Nuthetal, Germany; 4 German Center for Diabetes Research (DZD), Neuherberg, Germany; 5 Institute of Nutritional Science, University of Potsdam, Potsdam, Germany; 6 Department of Food Safety, German Federal Institute for Risk Assessment, Berlin, Germany; 7 Institute for Social Medicine, Epidemiology and Health Economics, Charité University Medical Center, Berlin, Germany; 8 Institute of Epidemiology, Christian Albrechts University Kiel, Kiel, Germany; 9 Department of Clinical Chemistry and Pathobiochemistry, Otto von Guericke University Magdeburg, Magdeburg, Germany; 10 Division of Endocrinology, Diabetology, Nephrology, Vascular Disease and Clinical Chemistry, Department of Internal Medicine, University of Tübingen, Tübingen, Germany; 11 Department of Epidemiology, German Institute of Human Nutrition Potsdam–Rehbrücke (DIfE), Nuthetal, Germany

## Abstract

**Importance:**

Inflammatory processes have been suggested to have an important role in colorectal cancer (CRC) etiology. Chemerin is a recently discovered inflammatory biomarker thought to exert chemotactic, adipogenic, and angiogenic functions. However, its potential link with CRC has not been sufficiently explored.

**Objective:**

To evaluate the prospective association of circulating plasma chemerin concentrations with incident CRC.

**Design, Setting, and Participants:**

Prospective case-cohort study based on 27 548 initially healthy participants from the European Prospective Investigation Into Cancer and Nutrition (EPIC)–Potsdam cohort who were followed for up to 16 years. Baseline study information and samples were collected between August 23, 1994, and September 25, 1998. Recruitment was according to random registry sampling from the geographical area of Potsdam, Germany, and surrounding municipalities. The last date of study follow-up was May 10, 2010. Statistical analysis was conducted in 2018.

**Main Outcomes and Measures:**

Incident CRC, colon cancer, and rectal cancer. Baseline chemerin plasma concentrations were measured by enzyme-linked immunosorbent assay.

**Results:**

A random subcohort of 221 incident CRC cases and 2329 participants free of CRC with available blood sample measurements were included in the analysis. The participants’ mean (SD) age was 50 (9) years, 62.1% were female, and 16.5% had a body mass index greater than 30. In multivariable-adjusted Cox proportional hazards regression models taking into account established CRC risk factors, higher chemerin concentrations were associated with a greater risk of CRC, with a hazard ratio (HR) of 1.81 (95% CI, 1.08-3.05; *P* for trend = .007) for the highest chemerin quartile vs the lowest. Analyses by cancer subsite indicated a stronger association with colon cancer (HR, 2.27; 95% CI, 1.18-4.34 for the highest quartile vs the lowest; *P* for trend = .005) compared with rectal cancer (HR, 1.27; 95% CI, 0.57-2.85; *P* for trend = .35). The association was particularly strong for proximal colon cancer (HR, 3.97; 95% CI, 1.51-10.50; *P* for trend = .001).

**Conclusions and Relevance:**

This study found that the association between chemerin concentration and the risk of incident CRC was linear and independent of established CRC risk factors. Further studies are warranted to evaluate chemerin as a novel immune-inflammatory agent in colorectal carcinogenesis.

## Key Points

**Question:**

Is there an association between peripheral concentrations of the proinflammatory biomarker chemerin and incident colorectal cancer?

**Findings:**

In this case-cohort study that included 221 incident colorectal cancer cases and 2329 cancer-free participants, higher circulating plasma chemerin concentration was associated with a greater risk of colorectal cancer. This association was independent of known risk factors for colorectal cancer, including age, adiposity, lifestyle, and metabolic factors.

**Meaning:**

Chemerin could have a role as an important inflammatory agent in the development of colorectal cancer and may serve as a promising future preventive and/or therapeutic target.

## Teaser

This case-cohort study evaluates the association of circulating plasma chemerin concentrations with incident colorectal cancer among adults in the European Prospective Investigation Into Cancer and Nutrition (EPIC)–Potsdam cohort.

## Introduction

Inflammation is considered 1 of the 7 hallmarks of cancer.^1^ Despite that the link between inflammation and cancer was proposed by Rudolf Virchow almost 2 centuries ago, the molecular and cellular mechanisms mediating this association remain unresolved.^2^ Both intrinsic and extrinsic pathways have been posited to explain this link. On the one hand, activation of oncogenes could promote construction of an inflammatory milieu; on the other hand, inflammatory conditions could contribute to the development of cancer.^3^ The colon may be particularly prone to proinflammatory carcinogenesis because of rapidly dividing cells and the presence of microbial flora, exposing colon mucosa to persistent low-grade inflammation.^4^ Inflammatory bowel disease, reflecting local inflammation of the colon, has been associated with increased colorectal cancer (CRC) risk,^5^ whereas the use of nonsteroidal anti-inflammatory drugs has been shown to lower CRC risk.^6^ Further proinflammatory triggers found to increase CRC risk or progression include environmental carcinogens (tobacco, alcohol, and diet) and metabolic conditions, such as obesity and insulin resistance.^5,7,8^ To date, several epidemiological studies^9,10,11^ have investigated associations between circulating biomarkers of inflammation and CRC risk, but the evidence has been inconclusive. Previous studies have generally focused on C-reactive protein (CRP), a nonspecific biomarker of chronic low-grade inflammation, whereas data on more specific immune-inflammatory molecules has been scarce.^12^ The term *inflammation* is broadly used to represent various pathophysiological processes orchestrated by biological mediators, including transcription factors, cytokines, chemokines, and infiltrating leukocytes.^13^ Chemokines have been suggested to link intestinal injury, inflammation, and cancer.^14,15,16,17^ Therefore, we identified chemerin as a promising immune-inflammatory molecule involved in the recruitment of immune cells to sites of injury.^18,19^ Chemerin is secreted as an 18-kDa preprotein form with low activity that undergoes further enzymatic proteolysis, acting as a chemoattractant for various cells involved in innate and adaptive immunity.^20,21^ Basal peripheral chemerin is reported to be largely secreted in adipose tissue and liver,^22^ and higher chemerin concentrations have been observed in people with obesity, metabolic conditions, and in those at a higher cardiovascular risk.^23,24,25^ In the context of cancer etiology, chemerin has been linked to tumor growth, angiogenesis, and metastasis.^26,27^ Higher concentrations of chemerin were found in patients with CRC compared with controls in a small case-control study^28^; however, prospective epidemiological studies on the link between chemerin concentrations and CRC are lacking. Therefore, we aimed to investigate the association of circulating chemerin concentrations with incidence of CRC using data from a large population-based prospective cohort study.

## Methods

### Study Population

**Figure 1.**
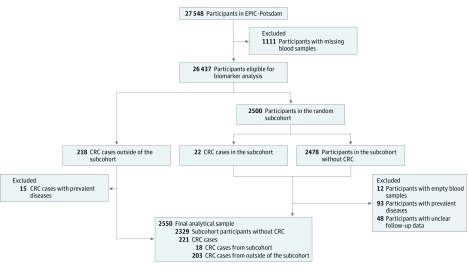
Flow Diagram Showing Study Sample Derivation CRC indicates colorectal cancer; EPIC, European Prospective Investigation Into Cancer and Nutrition.

This prospective case-cohort study was based on 27 548 initially healthy participants with available blood sample measurements from the European Prospective Investigation Into Cancer and Nutrition (EPIC)–Potsdam cohort who were followed for up to 16 years. The EPIC-Potsdam is a cohort study intended to prospectively investigate the role of diet and the development of cancer and other diseases.^29^ Participants were recruited between August 23, 1994, and September 25, 1998. Recruitment was according to random registry sampling from the geographical area of Potsdam, Germany, and surrounding municipalities. The overall study sample included 16 644 women and 10 904 men aged 35 to 64 years.^30^ Individuals were excluded from analyses if they reported a prior diagnosis of CRC or prevalent CVD (Figure 1). The association between chemerin concentration and incidence of CRC was evaluated using a case-cohort study design^31^ with blood samples from a random subcohort of EPIC-Potsdam participants. Overall, 221 incident CRC cases eligible for analysis were identified over a median follow-up of 10.6 years (interquartile range, 9.9-11.6 years), and 2329 participants free of CRC served as a control population in the randomly drawn subcohort. Information regarding incident disease diagnosis was obtained using disease and death registries and follow-up questionnaires sent every 2 to 3 years by mail. In the event of participant death, questionnaires were completed by the nearest relative. To verify disease status, once a participant was identified as a potential case, a standard inquiry form was sent to the treating physician and then evaluated by study physicians. The following *International Statistical Classification of Diseases*, *10th Revision* codes were used: carcinomas of the proximal colon (codes C18.0-18.5), distal colon (codes C18.6 and C18.7), and rectum (codes C19 and C20). Follow-up was defined as the time between enrollment and study exit, which was determined by diagnosis of the respective disease, death, dropout, or the censoring date, whichever occurred first. The last date of study follow-up was May 10, 2010. Statistical analysis was conducted in 2018.

 The study protocol was approved by the ethics committee of the Medical Society of the State of Brandenburg, Germany, and all participants provided written informed consent before enrollment. This study followed the Strengthening the Reporting of Observational Studies in Epidemiology (STROBE) reporting guidelines.^32^

### Data Collection and Laboratory Analysis

Baseline examination included detailed self-administered questionnaires about lifestyle, diet, and socioeconomic information, as well as computer-assisted interviews, measurement of anthropometric variables and blood pressure, and blood sample collection. Blood samples were stored in tanks of liquid nitrogen at −196°C or in deep freezers at −80°C until time of analysis. Anthropometric assessment was performed at study baseline by trained personnel.

Chemerin was measured in citrate plasma with a commercially available sandwich enzyme-linked immunosorbent assay (RD 191136200R; BioVendor Research and Diagnostic Products GmbH) at the Institute for Clinical Chemistry and Pathobiochemistry, Otto von Guericke University Magdeburg in 2015. The intra-assay coefficients of variation (CVs) ranged from 5.1% to 7.0%, the interassay CVs ranged from 6.9% to 8.3%, and the lower limit of detection was 0.1 ng/mL according to the manufacturer (BioVendor Research and Diagnostic Products GmbH). Based on our data, the interassay CV was 23%. Chemerin measurements are stable over a 4-month period (intraclass correlation coefficient, 0.72; 95% CI, 0.65-0.78) as previously reported.^24^ Plasma concentrations of high-density lipoprotein cholesterol (HDL-C) and high-sensitivity CRP (hsCRP) and red blood cell levels of glycated hemoglobin A_1c_ (HbA_1c_) were measured at the Department of Internal Medicine, University of Tübingen, with an automatic analyzer (ADVIA 1650; Siemens Medical Solutions).^33^ All biomarker measurements conducted in citrate plasma were corrected for the dilution introduced by citrate volume to improve comparability with other reported concentrations measured in EDTA plasma.^34^ Laboratory measurements were conducted by experienced technical personnel per the manufacturers’ instructions (Siemens Medical Solutions and Biovendor Research and Diagnostic Products GmbH). Missing values for body mass index (BMI) (2 cases and 0 noncases), HbA_1c_ (7 cases and 87 noncases), HDL-C (7 cases and 20 noncases), and hsCRP (7 cases and 15 noncases) were imputed using sex- and disease-specific median values.

### Statistical Analysis

Baseline characteristics among participants of the subcohort were evaluated according to quartiles of chemerin distribution. Cox proportional hazards regression analyses were used to estimate hazard ratios (HRs) and 95% CIs for the associations of chemerin with incident CRC. To account for the case-cohort study design, weights were assigned using the approach proposed by Prentice,^31^ and robust variance estimators were used to calculate 95% CIs using the methods described by Lin and Wei.^35^ To validate the appropriateness of the proportional hazards assumption, Schoenfeld residuals were calculated and assessed.

In Cox proportional hazards regression analyses, the associations were evaluated modeling chemerin categorically (according to quartiles of chemerin distribution in the subcohort) and continuously (per doubling of chemerin concentrations). Restricted cubic splines with 3 knots fitted at the 10th, 50th, and 90th percentiles of chemerin distribution were further used to evaluate the shape of the association, and the cubic spline and linear models were compared using likelihood ratio test. In multivariable-adjusted analyses, the models included the following covariates: age, sex, education, alcohol intake, smoking, physical activity, and dietary factors associated with CRC a priori, chosen based on the World Cancer Research Fund/American Institute for Cancer Research expert report,^36,37^ as well as BMI, waist circumference, and biomarkers associated with CRC^10^ (HbA_1c_, HDL-C, and hsCRP). For these analyses, a variable for waist circumference residually adjusted for BMI was used to account for potential overadjustment or collinearity. In additional analyses, the associations were explored according to anatomical subsites (distal colon cancer, proximal colon cancer, and rectal cancer). To account for competing risks, multivariable joint Cox proportional hazards regression models were used as described by Lunn and McNeil,^38^ and heterogeneity across sites was tested using Wald test. Kaplan-Meier survival analysis adjusted for the covariates listed above was performed to compare the onset of CRC among quartiles of chemerin distribution.

The associations were further evaluated according to subgroups by age, sex, alcohol intake, smoking, BMI, waist circumference, HbA_1c_, HDL-C, and hsCRP. Modification of the chemerin-disease associations by different levels of covariates was evaluated by introducing interaction terms and using Wald test to assess whether the slope for interaction differed from zero. We have been particularly interested in evaluating potential interaction (on the multiplicative and additive scale) with hsCRP as an established inflammatory biomarker using an approach described by Knol and VanderWeele.^39^

Finally, to explore bias in hazard ratios that could be introduced by reverse causality driven by subclinical inflammation and preclinical tumor formation, a detailed evaluation was performed according to different follow-up periods (3, 6, 10, and 12 years). In sensitivity analyses, individuals with the following characteristics were excluded from the analyses: recent or current flu or cold (n = 240), current aspirin use (n = 221), any prevalent cancer except nonmelanoma skin types (n = 146), prevalent type 2 diabetes (n = 108), hsCRP of 10 mg/L or higher (n = 89), or extreme chemerin concentration (below the 1st or above the 99th percentile [n = 51]) (to convert hsCRP level to nanomoles per liter, multiply by 9.524). Analyses were repeated in a subset of participants with available information on family history of CRC (n = 2333). Among the 177 participants with reported family history, there were 16 CRC cases (9.0%). The family history variable was modeled as a covariate in adjustment models. All statistical analyses were performed using a software program (SAS, version 9.4; SAS Institute Inc). A 2-sided *P* < .05 was considered statistically significant.

## Results

Among the random subcohort of 221 incident CRC cases and 2329 participants free of CRC with available blood sample measurements, the mean (SD) age was 50 (9) years, 62.1% were female, and 16.5% had a BMI (calculated as weight in kilograms divided by height in meters squared) greater than 30. The median chemerin concentration was 147.9 ng/mL (range, 49.9-368.5 ng/mL). The median chemerin concentration was 148.9 ng/mL (range, 49.9-368.5 ng/mL) for women and 146.7 ng/mL (range, 70.2-294.6 ng/mL) for men (*P* for difference = .13).

Higher concentrations of chemerin were observed in participants who were on average older, had greater BMI and waist circumference, and consumed higher quantities of red and processed meat and lower quantities of dairy products (eTable 1 in the Supplement). Concentrations of hsCRP were higher across categories of chemerin, whereas concentrations of HDL-C were lower.

In age- and sex-adjusted models, higher plasma chemerin concentrations were associated with a greater risk of CRC (HR, 2.10; 95% CI, 1.30-3.37 for the highest chemerin quartile vs the lowest; *P* for trend <.001) (Table 1). Further adjustment for education and established CRC risk factors (alcohol intake, smoking, and physical activity), BMI and waist circumference (residually adjusted for BMI), and the biomarkers HbA_1C_, HDL-C, and hsCRP did not markedly alter the observed associations (HR, 1.81; 95% CI, 1.08-3.05; *P* for trend = .007). In the final multivariable-adjusted model, a stronger association could be seen in women (HR, 2.40; 95% CI, 1.07-5.42; *P* for trend = .01) compared with men (HR, 1.41; 95% CI, 0.70-2.86; *P* for trend = .18); however, this difference was not statistically significant (*P* = .61).

**Table 1.** Combined and Sex-Stratified Multivariable-Adjusted Hazard Ratios (95% CIs) for CRC According to Quartiles of Chemerin Distribution and per Doubling in Chemerin Concentrations^a^

Chemerin by Sex and Overall	Adjusted Hazard Ratio (95% CI) for CRC by Chemerin Distribution Quartile					Hazard Ratio (95% CI) per Doubling of Chemerin
	Quartile 1	Quartile 2	Quartile 3	Quartile 4	*P* Value for Trend^b^	
Chemerin, median (IQR), ng/mL	111.6 (101.8-118.6)	136.2 (130.3-141.8)	157.3 (152.3-163.9)	192.5 (180.8-210.4)	NA	NA
**CRC Overall**						
No. of cases/noncases	26/586	45/580	68/579	82/584	NA	221/2329
Age- and sex-adjusted model	1 [Reference]	1.29 (0.78-2.12)	2.00 (1.24-3.22)	2.10 (1.30-3.37)	<.001	2.11 (1.35-3.29)
Model 2	1 [Reference]	1.19 (0.71-1.99)	1.94 (1.18-3.19)	1.97 (1.19-3.25)	.001	2.04 (1.25-3.31)
Model 3	1 [Reference]	1.17 (0.69-1.96)	1.79 (1.08-2.96)	1.81 (1.08-3.05)	.007	1.81 (1.09-3.00)
**CRC by Men**						
No. of cases/noncases	17/227	29/228	40/210	44/214	NA	130/879
Age- and sex-adjusted model	1 [Reference]	1.22 (0.66-2.28)	1.84 (1.00-3.39)	1.85 (1.00-3.43)	.02	2.05 (1.06-3.95)
Model 2	1 [Reference]	1.02 (0.53-1.97)	1.51 (0.79-2.88)	1.63 (0.83-3.21)	.07	2.03 (0.94-4.37)
Model 3	1 [Reference]	0.92 (0.47-1.81)	1.27 (0.65-2.47)	1.41 (0.70-2.86)	.18	1.72 (0.78-3.80)
**CRC by Women**						
No. of cases/noncases	9/359	16/352	28/369	38/370	NA	91/1450
Age- and sex-adjusted model	1 [Reference]	1.32 (0.58-3.02)	2.14 (0.98-4.66)	2.47 (1.15-5.29)	.005	2.37 (1.28-4.40)
Model 2	1 [Reference]	1.28 (0.56-2.94)	2.09 (0.95-4.59)	2.46 (1.12-5.41)	.006	2.61 (1.31-5.20)
Model 3	1 [Reference]	1.32 (0.57-3.06)	2.05 (0.92-4.58)	2.40 (1.07-5.42)	.01	2.54 (1.23-5.25)

Abbreviations: CRC, colorectal cancer; IQR, interquartile range; NA, not applicable.

^a^ Continuous log-transformed chemerin concentrations by log 2. Model 2 includes age, sex, education, alcohol intake, smoking, physical activity, dietary factors (fruit, fish, fiber, dairy products, red and processed meat, whole-grain bread, and nonstarchy vegetables), body mass index, and waist circumference residually adjusted for body mass index. Model 3 is model 2 plus high-density lipoprotein cholesterol, hemoglobin A_1c_, and high-sensitivity C-reactive protein. *P* for interaction between sex and chemerin = .61.

^b^ *P* value for trend from a linear model, calculated using the median chemerin concentration within quartiles as a continuous variable.

**Figure 2.**
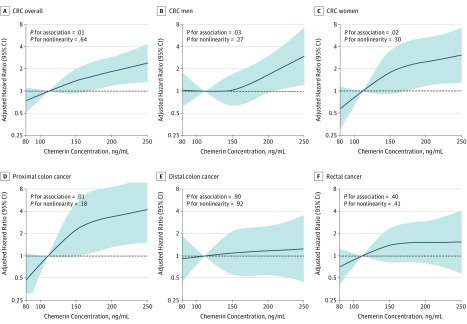
Multivariable-Adjusted Hazard Ratios for Colorectal Cancer (CRC) According to Chemerin Concentration Overall and by Sex and Cancer Subsite Hazard ratios and 95% CIs (shaded areas) were calculated by restricted cubic spline regression based on multivariable-adjusted model, including age, sex, education, alcohol intake, smoking, physical activity, dietary factors (fruit, fish, fiber, dairy products, red and processed meat, whole-grain bread, and nonstarchy vegetables), body mass index, and waist circumference residually adjusted for body mass index. Knots were placed at the 10th, 50th, and 90th percentiles. The median of the lowest chemerin concentration category (111.6 ng/mL) served as reference.

In restricted cubic spline Cox proportional hazards regression analyses, chemerin concentrations were positively associated with the risk of CRC, and no deviation from linearity could be seen (Figure 2). When modeled on the continuous scale, the respective HRs after final multivariable adjustment were 1.81 (95% CI, 1.09-3.00) for CRC overall and 1.72 (95% CI, 0.78-3.80) in men and 2.54 (95% CI, 1.23-5.25) in women (Table 1).

**Figure 3.**
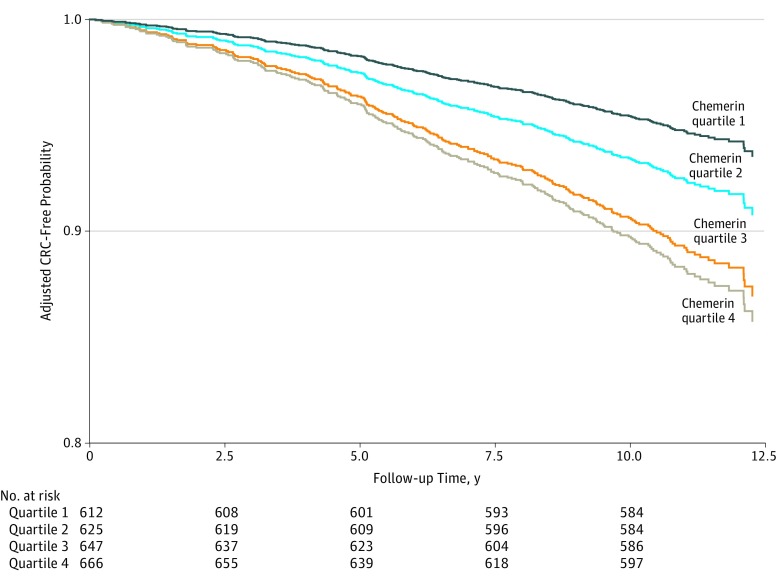
Kaplan-Meier Analysis for Survival Free of Colorectal Cancer (CRC) by Quartile of Chemerin Distribution Based on multivariable-adjusted model, including age, sex, education, alcohol intake, smoking, physical activity, dietary factors (fruit, fish, fiber, dairy products, red and processed meat, whole-grain bread, and nonstarchy vegetables), body mass index, and waist circumference residually adjusted for body mass index, high-density lipoprotein cholesterol, hemoglobin A_1c_, and high-sensitivity C-reactive protein.

Results from Kaplan-Meier analysis for survival free of CRC supported that higher chemerin concentrations were associated with decreased survival probability compared with lower concentrations. These findings are shown in Figure 3.

When analyses were conducted according to CRC anatomical subsites, a stronger association was observed for colon cancer (HR, 2.27; 95% CI, 1.18-4.34 in the final multivariable-adjusted model for the highest chemerin quartile vs the lowest; *P* for trend = .005) compared with rectal cancer (HR, 1.27; 95% CI, 0.57-2.85; *P* for trend = .35). However, this difference was not statistically significant (*P* for difference = .42) (Table 2).

**Table 2.** Multivariable-Adjusted Hazard Ratios (95% CIs) for Distal Colon Cancer, Proximal Colon Cancer, and Rectal Cancer According to Quartiles of Chemerin Distribution and per Doubling of Chemerin Concentrations^a^

Chemerin by Sex and Overall	Adjusted Hazard Ratio (95% CI) for Cancer by Chemerin Distribution Quartile					Hazard Ratio (95% CI) per Doubling of Chemerin
	1	2	3	4	*P* Value for Trend^b^	
Chemerin, median (IQR), ng/mL	111.6 (101.8-118.6)	136.2 (130.3-141.8)	157.3 (152.3-163.9)	192.5 (180.8-210.4)	NA	NA
**Colon Cancer Overall**^**c**^						
No. of cases/noncases	15/586	26/582	38/583	58/584	NA	137/2335
Age- and sex-adjusted model	1 [Reference]	1.34 (0.71-2.51)	1.92 (1.03-3.56)	2.56 (1.42-4.64)	<.001	2.45 (1.43-4.19)
Model 2	1 [Reference]	1.23 (0.64-2.37)	1.79 (0.94-3.44)	2.30 (1.22-4.35)	.003	2.17 (1.19-3.95)
Model 3	1 [Reference]	1.24 (0.64-2.39)	1.74 (0.90-3.36)	2.27 (1.18-4.34)	.005	2.11 (1.13-3.91)
**Proximal Colon Cancer**^**c**^						
No. of cases/noncases	6/586	11/584	14/586	33/586	NA	64/2342
Age- and sex-adjusted model	1 [Reference]	1.45 (0.55-3.84)	1.82 (0.69-4.79)	3.96 (1.64-9.55)	<.001	3.85 (1.90-7.79)
Model 2	1 [Reference]	1.40 (0.51-3.85)	1.83 (0.67-5.04)	3.96 (1.51-10.40)	<.001	3.80 (1.69-8.52)
Model 3	1 [Reference]	1.40 (0.51-3.89)	1.82 (0.66-5.04)	3.97 (1.51-10.50)	.001	3.96 (1.74-8.98)
**Distal Colon Cancer**^**c**^						
No. of cases/noncases	9/586	14/583	22/585	23/586	NA	68/2340
Age- and sex-adjusted model	1 [Reference]	1.11 (0.48-2.53)	1.78 (0.81-3.95)	1.58 (0.71-3.51)	.15	1.61 (0.74-3.51)
Model 2	1 [Reference]	0.92 (0.38-2.18)	1.55 (0.66-3.62)	1.22 (0.53-2.79)	.41	1.22 (0.53-2.82)
Model 3	1 [Reference]	0.90 (0.38-2.14)	1.45 (0.61-3.46)	1.16 (0.49-2.75)	.52	1.15 (0.47-2.82)
**Rectal Cancer**						
No. of cases/noncases	11/586	18/583	30/584	24/588	NA	83/2341
Age- and sex-adjusted model	1 [Reference]	1.22 (0.57-2.62)	2.08 (1.01-4.25)	1.44 (0.68-3.04)	.19	1.58 (0.77-3.21)
Model 2	1 [Reference]	1.19 (0.53-2.68)	2.27 (1.08-4.79)	1.49 (0.68-3.24)	.15	1.76 (0.84-3.72)
Model 3	1 [Reference]	1.10 (0.48-2.50)	1.95 (0.91-4.16)	1.27 (0.57-2.85)	.35	1.41 (0.65-3.06)

Abbreviations: IQR, interquartile range; NA, not applicable.

^a^ Continuous log-transformed chemerin concentrations by log 2. Model 2 includes age, sex, education, alcohol intake, smoking, physical activity, dietary factors (fruit, fish, fiber, dairy products, red and processed meat, whole-grain bread, and nonstarchy vegetables), body mass index, and waist circumference residually adjusted for body mass index. Model 3 is model 2 plus high-density lipoprotein cholesterol, hemoglobin A_1c_, and high-sensitivity C-reactive protein.

^b^ *P* value for trend from a linear model, calculated using the median chemerin concentration within quartiles as a continuous variable.

^c^ Overlapping lesions of colon (*International Classification of Diseases for Oncology*, *Third Edition* code C18.8) were not assigned a location. Therefore, distal and proximal colon cancer cases do not sum to the total colon cancer category.

In analyses by colon cancer subtype, higher chemerin concentrations were associated with a greater risk of proximal colon cancer (HR, 3.97; 95% CI, 1.51-10.50; *P* for trend = .001 for the final multivariable-adjusted model for the highest chemerin quartile vs the lowest), whereas no pronounced elevated risk was observed for distal colon cancer (HR, 1.16; 95% CI, 0.49-2.75; *P* for trend = .52) (Table 2). These differences by proximal and distal colon cancer were borderline statistically significant (*P* for difference = .08). A note of caution should be given regarding these results because of the fewer cases in the stratified analyses, leading to less precise HRs.

Overall, no substantial differences according to subgroups of study participants by age, adiposity, and metabolic status could be detected, and no clear effect modifiers jointly altering the observed associations were identified (eTable 2 in the Supplement). When analyses were stratified according to subgroups of hsCRP, higher chemerin concentrations were associated with a greater risk of CRC even in participants with low and moderate levels of hsCRP (eFigure 1 in the Supplement). For example, HRs for participants with low (<1 mg/L) and moderately high (1-3 mg/L) hsCRP and high chemerin concentrations (median, 192.5 ng/mL for quartile 4) were 1.90; 95% CI, 0.81-2.56 and 2.41; 95% CI, 1.48-4.72, respectively, compared with those with low hsCRP and low chemerin concentrations. No statistically significant interaction on either the multiplicative (HR of product term) or additive (relative excess risk due to interaction) scale was observed. When the associations were examined according to different follow-up times, no substantial differences in the HRs were found (eFigure 2 in the Supplement).

In sensitivity analyses, the observed associations were not substantially altered by exclusion of participants with the following characteristics: recent or current flu or cold, current aspirin use, any prevalent cancer except nonmelanoma skin types, prevalent type 2 diabetes, elevated hsCRP (≥10 mg/L), or extreme chemerin concentration. These results are summarized in eTable 3 in the Supplement. The overall trend of the associations was not changed after additional adjustment for family history of CRC.

## Discussion

In this prospective case-cohort study, chemerin concentrations were positively linearly associated with the risk of CRC. Overall, the associations were present in both men and women and were independent of participants’ age, adiposity, metabolic status, lifestyle, and baseline CRP levels, which are known to be associated with CRC risk. Furthermore, the association was present across low levels of CRP, suggesting a strong potential of chemerin concentration to reflect elevated risk beyond established inflammatory markers. Analyses by cancer subsite demonstrated that the associations were somewhat stronger for colon cancer compared with rectal cancer. The elevated risk was particularly pronounced for participants who developed proximal colon cancer compared with those who developed distal colon cancer.

Several lines of evidence support the role of systemic inflammation in colorectal carcinogenesis. The conversion of adenoma cells to adenocarcinoma cells in colon tissue is largely driven by inflammatory stimuli.^40,41^ Despite that, evidence on the etiological role of inflammatory biomarkers in the development of CRC has been inconclusive. Previous research largely focused on CRP as a single inflammatory biomarker, and evidence for additional inflammatory molecules has been scant.^42^ Several proinflammatory molecules secreted in adipose tissue and implicated in immune and metabolic pathways have also been associated with CRC, including adiponectin (particularly its non–high-molecular-weight form), soluble leptin receptor, and omentin.^43,44,45^ Our results showing a positive association between chemerin concentration and CRC support those lines of research implicating involvement of immune-inflammatory pathways in colorectal carcinogenesis. While the exact mechanisms explaining the biological association are unclear, several pathways could have a role and be consistent with systemically elevated chemerin concentrations. First, chemerin overexpression was shown to be associated with tumor angiogenesis^26^ and in experimental settings was observed to increase cancer cell invasiveness.^27^ Both functions represent mechanisms through which chemerin might exert direct influences on the development of cancer. Chemerin concentrations were also shown to be elevated in patients diagnosed as having CRC^28^ or gastric cancer.^46^ However, comparison of our results is limited due to retrospective study designs and small sample sizes of previous studies. Second, chemerin is involved in the recruitment of immune cells at the site of infection and could have a role as a proxy marker of systemic immune response.^47^ In particular, a link between bacterial infection and CRC was recently suggested by an analysis of 4063 incident cases of CRC matched to 4063 controls from 10 prospective cohorts.^48^ In that study, serologic responses to *Helicobacter pylori* proteins, including virulence factors VacA and CagA, were associated with a greater risk of CRC. Taking this evidence into account, we speculate that chemerin might act as a biomarker of pathogen-induced inflammatory response preceding tumorigenesis in the colon. Further studies are warranted to trace potential links between chemerin, bacterial and viral pathogenesis, and the development of CRC. Third, chemerin exerted regulatory functions in intestinal inflammation and was elevated in patients with inflammatory immune diseases, such as inflammatory bowel disease.^49^ Proinflammatory mediators are known to increase gut permeability, thereby inducing a local immune response and mucosal inflammation in the colon.^50^ Therefore, chemerin concentration could indicate colonic inflammatory response. However, we were unable to test such a hypothesis due to a lack of detailed data on gut permeability status in our study.

Fourth, in experimental models, chemerin expression increases dramatically with adipocyte development, which might translate into chronic inflammation and increased oxidative stress in obese individuals, who are at high risk of CRC.^51,52,53^ However, in our data, chemerin concentration was associated with an elevated risk of CRC independent of adiposity and CRP level. Furthermore, higher risk was also seen in individuals without obesity and with low to moderate hsCRP levels, favoring the hypothesis of more specific inflammatory pathways represented by chemerin concentration.

Fifth, chemerin concentration may merely represent subclinical inflammation associated with a tumor formation process that has already started. However, reverse causation due to early tumor formation in our data is unlikely because the associations persisted after exclusion of participants diagnosed as having cancer within the first years of study follow-up.

Our results by cancer subsite suggested particularly elevated risk for proximal colon cancer compared with distal colon cancer. While caution should be used in interpreting these results due to fewer participants in the stratified analyses, there may also be a biological explanation. The differences between the proximal colon and the distal colon in terms of developmental origin, environmental mutagens, and gut flora are increasingly recognized. Proximal carcinomas are more often mucinous and microsatellite unstable high, whereas distal carcinomas are more frequently human epidermal growth factor receptor 2 amplified and chromosome unstable.^54^ Concomitant with increased access to the mucosal epithelium, the microbial community forms a bacterial biofilm shown to be particularly present in the proximal colon.^55^ The direct bacterial contact with epithelial cells could result in perturbed epithelial function and chronic inflammation, thereby predisposing to proximal colon pathogenesis. Data from the Iowa Women’s Health Study^56^ suggested stronger associations between nonsteroidal anti-inflammatory drug use and proximal vs distal CRC. Future studies with large sample sizes are needed to have sufficient power to evaluate the observed associations according to CRC subsite.

### Strengths and Limitations

This study has several strengths. Our data are novel in providing first evidence on a prospective association between chemerin concentration and CRC risk. The analyses were based on a well-phenotyped study sample with a long follow-up time. Therefore, we were able to account for several factors that could potentially confound the observed associations and to evaluate potential reverse causality. We also explored the possible influence of effect modifiers, such as the inflammatory biomarker CRP, and found no evidence of statistical or biological interaction.

Our study also has some limitations. The study sample comprised participants within a prespecified age range and geographical residence. Replication studies are warranted to prove the validity of our findings in different population groups. Although we have accounted for some factors in our analyses, we did not have data on potentially important ones, such as detailed assessment of gut permeability and immune profiling. Further studies that assess potential interaction between chemerin and gut microbiota would provide essential information to test the raised hypotheses. Although we adjusted for many possible confounders, as in any observational study, we cannot also exclude the potential of residual confounding due to imprecise or missing measurements. For example, we accounted for dietary factors known to be prone to measurement error. We assessed chemerin as a single biomarker; however, data on additional inflammatory biomarkers (ie, cytokines, chemokines, and growth factors) could aid in characterizing associated pathophysiological pathways to a greater extent. In our study, a single blood sample was available for chemerin measurement, potentially introducing regression dilution bias; nevertheless, a reasonable midterm reliability of chemerin concentrations was observed over a 4-month period in a reproducibility study^24^ conducted before the present prospective cohort analysis. While we cannot provide data on the stability of chemerin in long-term storage at −80°C, it has been shown that freeze-and-thaw cycles do not substantially influence its measurements by the kit we used as per manufacturer protocol.

## Conclusions

To our knowledge, these are the first lines of evidence on a prospective association between circulating chemerin concentrations and CRC risk. The association was present in both men and women and was independent of age, adiposity, lifestyle and metabolic factors, and baseline CRP levels. Further studies are warranted to confirm our data and to elucidate pathways driving the observed associations.

## Notes

Supplement. **eTable 1.** Participant’s Characteristics of the EPIC-Potsdam Subcohort According to Quartiles of Chemerin Distribution **eTable 2.** Multivariable-Adjusted Hazard Ratios and 95% CIs for Colorectal Cancer per Doubling in Chemerin Concentrations According to Subgroups **eTable 3.** Sensitivity Analysis for the Association of Chemerin With Colorectal Cancer **eFigure 1.** Multivariable-Adjusted Hazard Ratios (95% CIs) for Colorectal Cancer According to Cross-Tabulated Chemerin and hsCRP Categories **eFigure 2.** Multivariable-Adjusted Hazard Ratios According to Different Follow-up Lengths
