# Supplementary material for: Association of Chemerin Plasma Concentration With Risk of Colorectal Cancer
Source: JAMA Netw Open. 2019 Mar 22;2(3):e190896. doi: 10.1001/jamanetworkopen.2019.0896 (PMC6583278; doi:10.1001/jamanetworkopen.2019.0896)
Supplement: Supplement. — eTable 1. Participant’s Characteristics of the EPIC-Potsdam Subcohort According to Quartiles of Chemerin Distribution eTable 2. Multivariable-Adjusted Hazard Ratios and 95% CIs for Colorectal Cancer per Doubling in Chemerin Concentrations According to Subgroups eTable 3. Sensitivity Analysis for the Association of Chemerin With Colorectal Cancer eFigure 1. Multivariable-Adjusted Hazard Ratios (95% CIs) for Colorectal Cancer According to Cross-Tabulated Chemerin and hsCRP Categories eFigure 2. Multivariable-Adjusted Hazard Ratios According to Different Follow-up Lengths [file jamanetwopen-2-e190896-s001.pdf]

Supplementary Online Content

Eichelmann F, Schulze MB, Wittenbecher C, et al. Association of chemerin plasma concentration with risk of colorectal cancer. *JAMA Netw Open*. 2019;2(3):e190896. doi:0.1001/jamanetworkopen.2019.0896

eTable 1. Participant's Characteristics of the EPIC-Potsdam Subcohort According to Quartiles of Chemerin Distribution

eTable 2. Multivariable-Adjusted Hazard Ratios and 95% CIs for Colorectal Cancer per Doubling in Chemerin Concentrations According to Subgroups

eTable 3. Sensitivity Analysis for the Association of Chemerin With Colorectal Cancer

eFigure 1. Multivariable-Adjusted Hazard Ratios (95% CIs) for Colorectal Cancer According to Cross-Tabulated Chemerin and hsCRP Categories

eFigure 2. Multivariable-Adjusted Hazard Ratios According to Different Follow-up Lengths

This supplementary material has been provided by the authors to give readers additional information about their work.

© 2019 Eichelmann F et al. *JAMA Network Open*.

eTable 1. Participants' Characteristics of the EPIC-Potsdam Subcohort According to Quartiles of Chemerin Distribution

	1st quartile	2nd quartile	3rd quartile	4th quartile
**Chemerin [ng/ml] median (range)**	111.6 (49.9 to 125.5)	136.2 (125.5 to 146.7)	156.9 (146.7 to 170.9)	192.5 (171.4 to 368.5)
**N**	586	585	588	588
**Socio-demographics**				
Age at recruitment [years], mean (SD)	46.6 (8.5)	50.0 (8.5)	50.3 (9.0)	53.3 (8.4)
Female sex, n (%)	358 (61.2)	350 (59.5)	373 (64.0)	372 (63.5)
University degree, n (%)	255 (43.6)	248 (42.2)	207 (35.5)	198 (33.8)
**Lifestyle**				
Physical activity [h/week], median (25th percentile to 75th percentile)	4.5 (2.5, 8.0)	5.0 (2.0, 8.5)	4.5 (2.0, 8.0)	4.5 (1.5, 8.5)
Alcohol [g/day], median (25th percentile to 75th percentile)	9.0 (3.3, 21.2)	9.0 (2.8, 21.0)	8.6 (2.7, 20.4)	7.8 (2.9, 18.6)
Current smoker, n (%)	129 (20.3)	113 (19.3)	133 (22.8)	125 (21.3)
**Anthropometry**				
Body mass index [kg/m²], mean (SD)	23.9 (3.1)	25.5 (3.3)	26.4 (4.2)	28.5 (4.8)
Waist circumference [cm], mean (SD)	79.6 (10.5)	84.2 (11.6)	86.6 (12.5)	92.3 (12.9)
Waist circumference in men	88.0 (8.4)	93.4 (8.4)	95.4 (9.6)	99.5 (9.9)
Waist circumference in women	74.3 (7.9)	78.1 (9.1)	81.4 (11.0)	88.2 (12.7)
**Dietary factors (WCRF report)**				
Fiber intake [g/day], median (25th percentile to 75th percentile)	21.4 (17.5, 26.5)	21.8 (17.9, 25.8)	21.1 (16.8, 25.8)	21.3 (17.5, 25.9)
Fruits [g/day], median (25th percentile to 75th percentile)	99.4 (82.5, 182.9)	101.5 (87.0, 188.5)	97.2 (77.5, 184.9)	98.7 (78.5, 190.2)
Vegetables [g/day], median (25th percentile to 75th percentile)	109.8 (82.6, 146.3)	117.1 (85.2, 155.7)	113.4 (80.5, 156.5)	119.8 (87.8, 159.0)
Red meat [g/day], median (25th percentile to 75th percentile)	34.5 (21.7, 49.5)	36.2 (22.9, 54.8)	38.7 (23.9, 58.1)	37.8 (25.5, 57.7)
Processed meat [g/day], median (25th percentile to 75th percentile)	48.7 (29.8, 75.3)	50.0 (30.4, 78.3)	51.1 (36.6, 78.3)	50.4 (29.4, 80.4)
Fish [g/day], median (IQR)	18.1 (9.9, 29.0)	18.9 (9.9, 29.0)	18.6 (9.9, 30.1)	18.4 (9.9, 29.0)
Dairy [g/day], median (25th percentile to 75th percentile)	180.8 (95.4, 285)	178.1 (103.2, 280.8)	163.7 (88.9, 272.8)	163.2 (95.2, 275.2)
**Biomarkers**				
HDL-C [mmol/L], median (25th percentile to 75th percentile)	1.5 (1.3, 1.7)	1.4 (1.2, 1.7)	1.4 (1.2, 1.7)	1.3 (1.1, 1.6)
hsCRP [mg/L], median (25th percentile to 75th percentile)	0.3 (0.1, 0.8)	0.6 (0.2, 1.4)	1.0 (0.4, 2.7)	1.8 (0.7, 4.5)
HbA_{1c} [%], median (25th percentile to 75th percentile)	5.4 (5.1, 5.7)	5.4 (5.1, 5.7)	5.4 (5.1, 5.7)	5.5 (5.3, 5.8)

Abbreviations: EPIC, European Prospective Investigation into Cancer and Nutrition; SD, standard deviation; WCRF, World Cancer Research Fund; HDL-C, high-density lipoprotein cholesterol; hsCRP, high-sensitivity c-reactive protein; HbA_{1c}, glycated hemoglobin

© 2019 Eichelmann F et al. *JAMA Network Open*.

eTable 2. Multivariable-Adjusted Hazard Ratios and 95% CIs for Colorectal Cancer per Doubling in Chemerin Concentrations According to Subgroups

	**Cases/ non-cases**	**HR (95%-CI)**	$P_{interaction}$[a]
**Age**			0.91
≤ 50 years	91/1450	2.48 (0.88, 6.95)	
> 50 years	130/879	1.98 (1.13, 3.46)	
**Smoking at baseline**			0.51
no	185/1840	2.02 (1.17, 3.49)	
yes	36/489	2.08 (0.50, 8.66)	
**Average alcohol intake**[b]			0.76
< 8.6 g/day	94/1181	2.19 (1.05, 4.57)	
≥ 8.6 g/day	127/1148	2.08 (1.05, 4.11)	
**Prevalent diabetes**			0.06
no	211/2231	2.29 (1.38, 3.82)	
yes	10/98	0.62 (0.37, 1.04)	
**BMI**			0.41
<30 kg/m²	170/1958	1.93 (1.14, 3.27)	
≥30 kg/m²	51/371	2.52 (0.74, 8.55)	
**Waist circumference**			0.74
< ♂94/♀80 cm	84/1217	2.30 (1.06, 4.99)	
≥ ♂94/♀80 cm	137/1112	2.08 (1.08, 4.00)	
**HDL-C**			0.50
< ♂1.04/♀1.30 mmol/L	149/1789	1.72 (0.94, 3.15)	
≥ ♂1.04/♀1.30 mmol/L	72/540	3.36 (1.35, 8.41)	
**HbA1c**			0.32
< 5.7%	147/1695	1.96 (1.08, 3.56)	
≥ 5.7%	74/634	2.54 (1.02, 6.29)	

Based on model 2 (age- and sex-adjusted model + education, alcohol intake, smoking, physical activity, dietary factors (fruit, fish, fiber, dairy, red and processed meat, whole grain bread, non-starchy vegetables), BMI, and waist circumference residually adjusted for BMI); [a] calculated by use of cross-product term between continuous (log2) chemerin concentration and categorized/dichotomous possible effect measure modifying variable; [b] population median as cut-point; Abbreviations: HDL-C, high-density lipoprotein cholesterol; HbA_{1c}, glycated hemoglobin

© 2019 Eichelmann F et al. *JAMA Network Open*.

eTable 3. Sensitivity Analysis for the Association of Chemerin With Colorectal Cancer

	**Cases/ non-cases**	**HR (95%-CI) per doubling**
Model 2	221/2329	2.04 (1.25, 3.31)
Exclusions due to:		
Extreme concentrations[a]	215/2284	1.87 (1.11, 3.14)
Recent/current flu/cold	204/2106	1.92 (1.17, 3.16)
hsCRP ≥10 mg/L	210/2251	2.14 (1.30, 3.50)
Current Aspirin use	199/2130	2.13 (1.29, 3.50)
Any prevalent cancer[b]	196/2208	1.92 (1.15, 3.19)
Prevalent Type 2 diabetes	211/2231	2.29 (1.38, 3.82)

Based on model 2 (age- and sex-adjusted model + education, alcohol intake, smoking, physical activity, dietary factors (fruit, fish, fiber, dairy, red and processed meat, whole grain bread, non-starchy vegetables), BMI, and waist circumference residually adjusted for BMI); [a] concentrations below 1st and above 99th percentile ; [b] e xcept non-melanoma skin cancer; Abbreviations: hsCRP, high-sensitivity c-reactive protein;

© 2019 Eichelmann F et al. *JAMA Network Open*.

eFigure 1. Multivariable-Adjusted Hazard Ratios (95% CIs) for Colorectal Cancer According to Cross-Tabulated Chemerin and hsCRP Categories

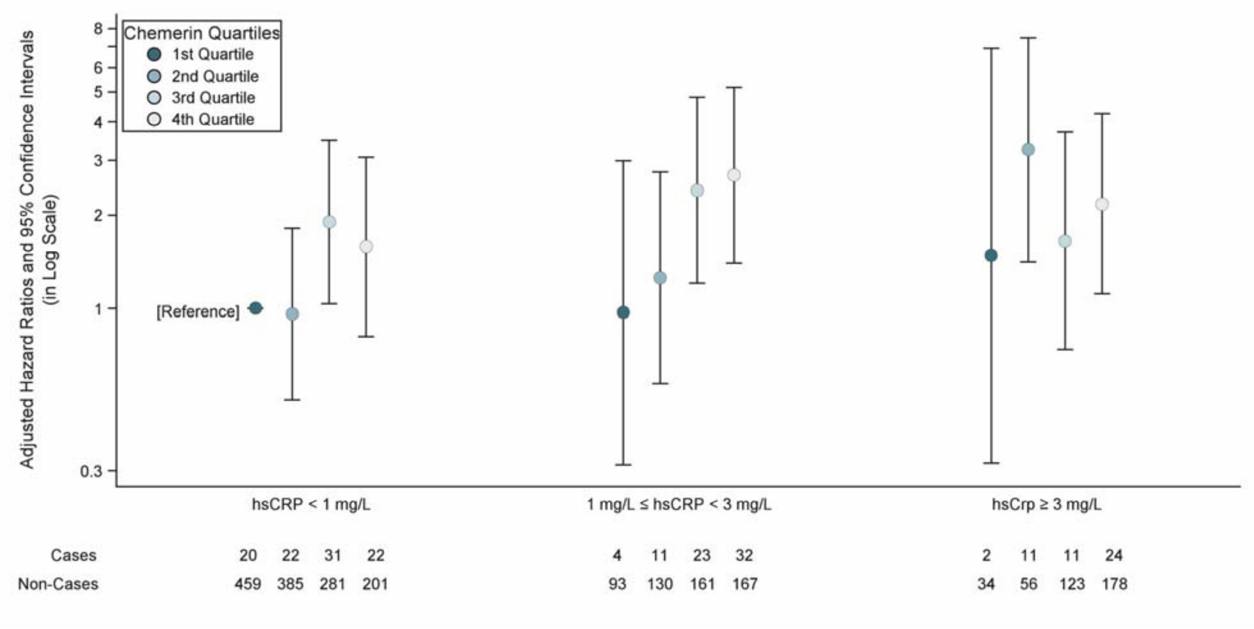

Hazard ratios and 95%-CI calculated by assigning participants groups according to chemerin distribution by quartiles and hsCRP categories (low: <1 mg/L, moderate: 1-<3 mg/L, high: ≥3 mg/L). Participants with extreme hsCRP (≥10 mg/L) concentrations were excluded for this analysis. Based on multivariable-adjusted model including age sex, education, alcohol intake, smoking, physical activity, dietary factors (fruit, fish, fiber, dairy, red and processed meat, whole grain bread, non-starchy vegetables), BMI, and waist circumference residually adjusted for BMI. Abbreviations: hsCRP, high-sensitivity C-reactive protein; BMI, body mass index; HR, hazard ratio;

© 2019 Eichelmann F et al. *JAMA Network Open*.

eFigure 2. Multivariable-Adjusted Hazard Ratios According to Different Follow-up Lengths

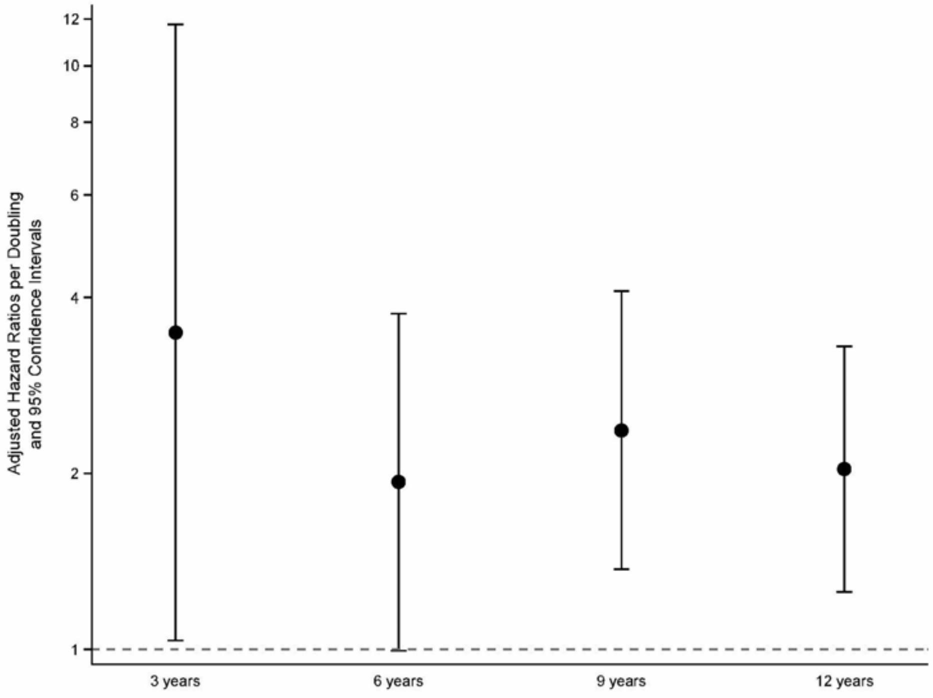

Based on multivariable-adjusted model including age sex, education, alcohol intake, smoking, physical activity, dietary factors (fruit, fish, fiber, dairy, red and processed meat, whole grain bread, non-starchy vegetables), BMI, and waist circumference residually adjusted for BMI.

© 2019 Eichelmann F et al. *JAMA Network Open*.
